# Digital Inclusion among Community Older Adults in the Republic of Korea: Measuring Digital Skills and Health Consequences

**DOI:** 10.3390/ejihpe14080154

**Published:** 2024-08-08

**Authors:** Thet Htoo Pan, Myo Nyein Aung, Eun Woo Nam, Yuka Koyanagi, Hocheol Lee, Li Li, Myat Yadana Kyaw, Nadila Mulati, Saiyud Moolphate, Carol Ma Hok Ka, Jan A. G. M. van Dijk, Motoyuki Yuasa

**Affiliations:** 1 Department of Global Health Research, Graduate School of Medicine, Juntendo University, Tokyo 113-8421, Japan; thet.fp@juntendo.ac.jp (T.H.P.); l.li.cj@juntendo.ac.jp (L.L.); myat.rr@juntendo.ac.jp (M.Y.K.); m.nadila.vp@juntendo.ac.jp (N.M.); moyuasa@juntendo.ac.jp (M.Y.); 2 Faculty of International Liberal Arts, Juntendo University, Tokyo 113-8421, Japan; 3 Advanced Research Institute for Health Sciences, Juntendo University, Tokyo 113-8421, Japan; 4 Department of Health Administration, Software Digital Healthcare Convergence College, Yonsei University, Wonju City 26493, Republic of Korea; ewnam@yonsei.ac.kr; 5 Department of Judo Therapy, Faculty of Health Sciences, Tokyo Ariake University of Medical and Health Sciences, Tokyo 135-0063, Japan; y-koyanagi@juntendo.ac.jp; 6 Department of Health Administration, Yonsei University Graduate School, Wonju City 26493, Republic of Korea; lhc0104@yonsei.ac.kr; 7 Department of Public Health, Faculty of Science and Technology, Chiang Mai Rajabhat University, Chiang Mai 50300, Thailand; saiyud_m@cmru.ac.th; 8 S R Nathan School of Human Development, Singapore University of Social Sciences, Singapore 599494, Singapore; carolmahk@suss.edu.sg; 9 Department of Communication Science, University of Twente, 7500 AE Enschede, The Netherlands; jan.vandijk@utwente.nl

**Keywords:** social gerontology, digital sociology, psychometric, mixed method, healthy aging, digital education, global health, public health, DIHAC, South Korea

## Abstract

Many older adults are increasingly embracing digital technology in the Republic of Korea. This study investigated the relationship between the digital skills of Korean older adults and their perceived health status and digital technology application for health promotion. This mixed-method study comprised a community survey of 434 older adults aged ≥65 in two cities in South Korea, followed by focus group interviews. Five types of digital skills, ‘operational internet skills’, ‘information navigation skills’, ‘social skills’, ‘creative skills’, and ‘mobile skills’, were measured using the LSE digital skill measurement instrument. Multivariable analysis identified the influence of digital skills on health-related outcomes. Among them, ‘social skills’ associated positively with self-rated health (β 0.37, 95%CI 0.08, 0.65). ‘Information navigation skills’ contributed positively to the use of digital technology and the internet for a healthy lifestyle in terms of improving eating habits (β 0.43, 95%CI 0.09, 0.77), accessing healthcare (β 0.53, 95%CI 0.21, 0.85), and accessing long-term care services (β 0.45, 95%CI 0.11, 0.79). Thematic analysis revealed that the study participants use Korean language-based resources such as Naver and Kakao Talk for social connection to promote a healthy lifestyle. This study concludes that encouraging initial and sustained use of the internet and enhancing digital skills among Korean older adults can promote active and healthy aging.

## 1. Introduction

Digital skills and internet connectivity have been called the “super social determinants of health” [1]. Daily activities are increasingly reliant on digital technologies and therefore digital skills are recognized as fundamental [2]. Digital inclusion mediates access to other social determinants of health directly, through access to and literacy in technologies or services, and indirectly, by supporting people’s capacity to engage fully and equitably in health promotion activities and social inclusion [3]. Therefore, developing the digital skills of older people can be both fulfilling and empowering, and leveraging these skills can pave the way to the goal of establishing digitally inclusive healthy aging communities.

Digital technology has been proven to have a positive impact on health and lifestyle behaviors. In particular, during the COVID-19 pandemic, digital technologies were key to combatting loneliness and isolation among older people [4]. Using the internet is associated with better physical and cognitive health, social well-being, health behaviors, and social support [5,6,7]. Moreover, aging in place can be achieved by improving the quality of life of older adults through innovation in digital health [8]. Recent studies on individuals with higher digital health literacy scores showed better self-management and participation in their own medical decisions, mental well-being, and quality of life [9]. There is a strong association between increasing digital skills for online information-seeking and subjective well-being in older adults [10]. Moreover, developing high e-health literacy and beliefs about medicine can improve medication adherence [11]. Therefore, eliminating the gray digital gaps may have the potential of promoting healthy aging and health equity. 

However, the full benefits of the internet and digital technology can be achieved only if people have access to the internet and acquire a basic level of digital skills. Approximately 5.4 billion people (67 percent) of the world’s population were using the internet in 2023, which leaves 2.6 billion people still offline [12]. For most of the cohort groups of older people, learning digital technology was not part of their formal education. Digital skills among older adults are heterogeneous, based on their experience of and exposure to internet digital technology throughout their life [13]. 

Research on minimizing the gray digital divide has become increasingly important, particularly in leveraging digital skills in those countries currently facing population aging. South Korea, formally known as the Republic of Korea (ROK), is the third most aging country in Asia and the Pacific region with 18.4% of the population aged 65 or over, which is expected to rise to 39.4% by 2050 [14]. The country is estimated to be the first country in the world with life expectancy exceeding 90 years [15]. On the other hand, South Korea recorded one of the lowest fertility rates in the world with 0.72 in 2023 [16]. Declining fertility rates and longer life expectancy lead to the rapid demographic transition towards population aging. The country is estimated to become a super-aged society by 2026 [17]. Alongside the aging population, digital technology is also rapidly advancing in South Korea. The COVID-19 pandemic has further accelerated Korea’s digitization efforts in areas such as information sharing, contact tracing, mHealth, e-education, and telework [18]. Internet penetration within the general population was 97.2% in 2022, much higher than the global average (66.3%) according to the International Telecommunication Union (ITU) [19]. 

Despite South Korea bridging the first level digital divide, internet penetration among older people has remained low. Forty-five percent of people aged over 70 reported not using the internet according to the 2022 Korean National Survey [20]. Among the four most vulnerable classes, the digital divide of older people was the largest in South Korea [21]. Older adults who stay disconnected from the internet could experience a growing gap in accessing vital online services including health information and health and social care services. As well as connectivity and internet access, individual digital skills are also important [22,23]. The inability to use the internet or having a low level of skill in using the internet, despite having access to it, is classified as the second level digital divide [24,25]. Such a divide can be common among older people, but has yet to be measured in many countries. Scholars’ attention towards digital inclusion among South Korean older adults has significantly increased in recent years, yet the inconsistency in measuring the level of digital skills among older adults in the ROK remains unresolved [26]. This study aimed to fill this gap in the literature in the Republic of Korea, where access to the internet is well established, yet the skill gap may remain amongst the older generation. 

Even though older adults have access to the internet, their digital skill gaps may leave them behind, such as being unable to participate in social activities, or use social services online [27,28,29]. Consequently, it can cause inequality in outcomes such as health promotion, healthcare service, education, and productivity. This is known as the third-degree digital divide [30]. Third-degree gaps among older people may be common but have not yet been reported in the setting of South Korea. In this study, the self-perceived health status of the participants, and their ability to use digital technology for health promotion, were set as participatory outcomes. Furthermore, we sought to investigate which particular type of digital skill relates to older adults’ ability to adopt a healthy lifestyle [31].

Therefore, the current study aimed to (i) measure the levels of digital skills of community resident older adults; (ii) identify their internet usage characteristics: internet access, time spent on the internet, types of digital devices, and SNS use; and (iii) investigate the association of different types of digital skills with participatory outcome in two aspects: (i) perceived health; and (ii) digital technology use for health promotion activities. In addition, we investigated why and how older South Korean adults use the internet and digital technology for active and healthy aging. 

## 2. Materials and Methods

A mixed-methodology, explanatory sequential design (Quan > qual) was conducted [32]. Quantitatively, 5 types of digital skills were investigated among Korean older adults and their association with self-rated health and digital technology use for health promotion activities. Qualitatively, how and why older adults in the Republic of Korea use digital technology for active and healthy aging was explored. This study is part of the ongoing study “Digitally Inclusive, Healthy Aging Communities (DIHAC): A Cross-Cultural Study in Japan, Republic of Korea, Singapore, and Thailand” [33]. Ethical approval for the DIHAC study was obtained from the Juntendo University Ethical Committee (approval number E22-0057-M01) and the Yonsei University Institutional Review Board (1041849-202304-SB-073-02). 

### 2.1. Quantitative Method

#### 2.1.1. Study Participants 

A community survey was conducted, recruiting participants in Yeoju-si, Gyeonggi-do, and Wonju-si, Gangwon-do, two cities in the ROK. The study site comprised cities and urban areas, but also accommodated older persons that resettled from rural villages. The inclusion criteria of the study participants were older residents aged 65 years and over, residing in a community that has ongoing community-based health promotion activities. Participants were recruited at community welfare centers for older adults during their visits to the centers in February 2023. The sample size was calculated to ensure a power of 80% and 95% confidence interval for the findings, based on the percentage of internet use among older people in the ROK, and was estimated to be 360 participants. The sample size calculation applied one sample estimation of proportion using Stata SE16. The number of samples was inflated by 20 percent, compensating for the non-responses [33]. Data collection was conducted through a face-to-face survey using a structured questionnaire translated into the native Korean language. Research assistants were trained before data collection. Written informed consent forms were obtained from all participants involved in the study. A pilot study and translation were properly conducted to ensure reliability and validity. A sample of 444 participants answered the survey questionnaire. After excluding 10 participants who were younger than 65, a total of 434 participants were analyzed, of which 72.58 percent were female. The mean age of the sample was 76.75 years (SD 6.55).

#### 2.1.2. Research Instruments

##### Socio-Demographic Characteristics

Socio-demographic characteristics include age, sex, education, and income [25,30,34]. Pension status was added due to the national pension service existing in the country context and all the study participants were over 65 years old. Two questionnaires on physical health problems related to aging were carried out since these can influence digital device usage [35,36]. “Eye problems when using a digital device such as a mobile phone or a computer”, and “hand problems when using a digital device such as a mobile phone or a computer (Ex. flexion deformity)”, categorized as Yes or No responses, were included.

##### Internet Usage Characteristics

Physical access to the internet includes having an internet environment and owning a digital device [23]. Types of internet environment at home were categorized as ‘Non-user’; ‘Mobile internet user’; ‘Fixed broadband (Fiber, ADSL) user’; and ‘Do not know what I am using’. Types of digital devices used were recorded as ‘Non-user’; ‘Smartphone user’; ‘Mobile phone user’; ‘Personal computer user’; and ‘Tablet user’. Types of Social Networking Services (SNS) used included ‘Non-user’; ‘Kakao Talk’; ‘YouTube’; ‘Facebook’; ‘Line’; ‘Messenger’; and ‘Other SNS’. Types of home internet environment, digital devices, and SNS use were mutually non-exclusive data, coded as dichotomy ‘Yes’ or ‘No’ responses, describing user numbers and percentages in tables. Digital devices and SNS use were recoded according to ownership status. To estimate the time spent on the internet, the number of hours per day and the number of days per week spent on the internet were recorded. Then, internet usage per week was calculated and classified into 4 groups: ‘Non-users (0 h)’; ‘Low users of the Internet (<4 h)’; ‘Regular users (4 to 24 h)’; and ‘Broad users (≥24 h)’ [37].

##### Digital Skill Measurement

The perceived digital skill of the study participants was measured using the “Digital skill measurement scale” of the London School of Economics and Political Science (LSE) and translated into a Korean version. It is a 22-item, 5-point Likert scale measuring five domains: (i) ‘operational internet skills’ (5 items); (ii) ‘information navigation skills’ (5 items); (iii) ‘communicational/social internet skills’ (5 items); (iv) ‘creative skills’ (5 items); and (v) ‘mobile internet skills’ (2 items) [30]. ‘Operational skills’ are the basic technical skills required to use the internet, often referred to as “button knowledge”. ‘Information navigation skills’ relate to searching for information including the ability to find, select, and evaluate sources of information on the internet. The scores were reversed since it contained negatively worded items. ‘Social skills’ measure the ability to use online communication and interactions, often called “netiquette”. ‘Creative skills’ are the skills needed to create content of acceptable quality to be published or shared with others on the internet. The high penetration of smartphones was also considered to be important; thus, a ‘mobile internet skills’ category was included. All questions had response options ranging from 1: ‘Not at all true of me’ to 5: ‘Very true of me’. The combination of all skills provided a holistic view of the ability to function effectively online. 

Transcultural translation of the LSE digital skill measurement scale was followed by forward translation, back translation, a cognitive test, and pilot study. The original English set of questionnaires including the LSE digital skill measurement scale was translated into Korean language by two independent native speaker researchers. The two translations, one each from the translators, were synthesized into a Korean language version by a panel of experts consisting of the translators and the Korean researcher. Backtranslation was conducted by an external expert. It was compared to the original English version to ensure that the concept of questions had not been lost in translation. The process of translation and adaptation of instruments was conducted according to WHO guidelines [38]. A pilot study was conducted to test the readability, comprehension and reliability of the instrument. Items were selected for the final set by the team. All 5 domains of the 22-itemed LSE digital skills questionnaires exhibited high internal consistency: operational internet skills (α = 0.98); information navigation skills (α = 0.94); social skills (α = 0.98); creative skills (α = 0.85); and mobile skills (α = 0.88).

##### Participatory Outcomes

Self-rated health status was measured by evaluating on the 4-point Likert scale ranging from 1 to 4: ‘Very healthy’ to ‘Not healthy’. Previous studies about self-rated health showed strong validity and reliability across various populations including older adults and the scale could predict the objective health status [39,40]. 

Digital technology use for health promotion activities was measured using three questionnaires. The frequency that participants used the internet and digital technology to (i) improve eating habits, (ii) access healthcare services, and (iii) access long-term care services was measured. Data were collected using a 5-point Likert scale ranging from 0 to 4: ‘Never’ to ‘Usually’ (α = 0.93).

#### 2.1.3. Data Analysis

The statistical analysis was performed using Stata/ SE 17.0 (StataCorp. 2021. Stata Statistical Software: Release 17. College Station, TX, USA: StataCorp LLC). Descriptive statistics include socio-demographic characteristics, internet usage characteristics, digital skills, self-rated health, and digital technology use for health promotion activities. Frequency and percentage describe categorical data and mean and standard deviation (Mean ± SD) for continuous data. The normality of the data was checked using the Shapiro–Wilk test. The distribution of digital skills across sociodemographic characteristics and digital characteristics were described by descriptive statistics (Mean ± SD). The difference in mean scores was analyzed by the two-sample Wilcoxon signed-rank test or Kruskal–Wallis’s equality-of-populations rank test. Ordinal logistic regression was used to find the association of digital skills with self-rated health and digital technology use for health promotion activities. The multivariable regression model treated age, gender, income, and education as covariates. A *p*-value < 0.05 defined statistical significance with 95% CI. The models were confirmed to be free from multicollinearity.

### 2.2. Qualitative Method

The study district was in Wonju-si City, the ROK. Interviews took place at three community centers for older adults where part of the quantitative data were collected. Information regarding the interviews was given to the participants during the quantitative survey. Twenty-six community-dwelling older adults were recruited using convenient sampling including those who had participated in the survey and those who had not. Three of the older adults were male and twenty-three were female. The age range was 65 to 94 years, representing diverse internet experiences. Four focus group interviews were conducted in January 2024 following the survey in 2023. The interviewers used semi-structured questionnaires based on the psychometric measurements in quantitative phase to seek to understand why older people use the internet and digital technology and how they applied different types of digital skills for active and healthy aging. Each interview lasted between 20 and 60 min and was audio-recorded for subsequent verbatim transcription and translation. Thematic analysis was conducted, with themes conceptualized by domain summaries [41]. Data were integrated throughout the research. 

## 3. Results

### 3.1. Quantitative Findings

#### 3.1.1. Socio-Demographic Characteristics of the Participants

A total of 434 participants were included in the study. The mean age was 76.75 ± 6.55 years. The sample was skewed towards younger cohorts: young-old (40.28%) and old-old groups (43.98%). More than 70% were female (N = 315, 72.58%). In terms of education, 28.57% of the participants were primary school graduates, whilst 16.13% did not go to school. A majority of the participants did not have an income (62.21%). Among those who had an income, 16.36% belonged to the first quartile (having income lower than or equal to KRW one million). At the time of data collection, 385 participants (88.71%) were receiving pension. Regarding physical problems, 65.59% reported having eyesight problems in using a digital device, whereas only 2.23% had hand problems such as flexion deformity (Table 1).

#### 3.1.2. Digital Skills and Internet Usage

Out of the five domains of digital skills, ‘social skills’ scored the highest (2.16 ± 1.61), followed by ‘information navigation skills’ (1.90 ± 1.36), ‘operational internet skills’ (1.77 ± 1.44), and ‘mobile skills’ (1.74 ± 1.29). ‘Creative skills’ were the lowest (1.23 ± 0.48) (Range: 1 to 5) (Table 2).

In relation to internet usage, 191 older adults (44.01%) reported having physical access to the internet. ‘Broadband’ was the type most commonly used in the home internet environment (31.41%), whilst 238 older adults (54.97%) were non-users of the internet in the home environment. Smartphone owners accounted for 47.47% of the participants, followed by personal computer owners (9.68%); 40.55% were single-device owners, whilst tablet owners had the highest digital ownership status having three devices (0.69%); 218 older adults (50.23%) did not use any device to access the internet (Table 2). Among the internet users, the median time spent on the internet was 7 h per week (Interquartile range 5–14). More than one-third were regular internet users who spent 4 to 24 h per week on the internet (33.17%). The most used SNS was Kakao Talk (37.79%), followed by YouTube (29.03%) and Facebook (5.30%). A very small number of participants used Line (2.76%), Facebook Messenger (0.23%), and Naver Band (0.23%). More than half of the participants did not use any social media (57.60%). One in four participants used two applications (25.12%), 13.36% were single application users, whilst 3.92% were three application users. (Table 2). 

Differences in the mean scores of digital skills were measured between socio-demographic backgrounds (Supplementary Table S1). Men scored higher in all domains: ‘operational skills’ (2.17 ± 1.72 vs. 1.61 ± 1.29), ‘information-navigation skills’ (2.24 ± 1.49 vs. 1.78 ± 1.28), ‘social skills’ (2.66 ± 1.80 vs. 1.97 ± 1.49), ‘creative skills’ (1.37 ± 0.58 vs. 1.18 ± 0.42), and ‘mobile skills’ (2.13 ± 1.51 vs. 1.59 ± 1.17). 

**Figure 1:**
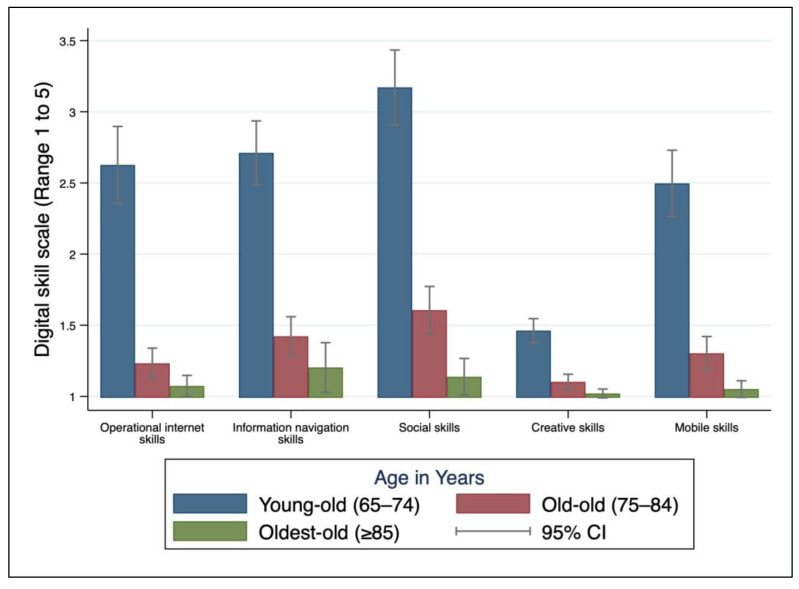
Distribution of five types of digital skills across age group among community older adults in the Republic of Korea (N = 434).

The oldest-old age group had lower digital skills than the old-old group, which in turn had lower skills than the young-old group in all types of digital skill (Figure 1). The lower the education level, the lower the digital skill scores. University graduates and higher scored the highest in all five domains. Digital skills varied by income. Older adults who had the second quartile of monthly income (KRW 1–2.5 million) scored highest in ‘operational skills’ (2.16 ± 1.65), ‘social skills’ (2.81 ± 1.74), and ‘mobile skills’ (2.09 ± 1.36). ‘Information navigation skills’ showed a statistical difference in relation to pension status (1.88 ± 1.36 vs. 2.09 ± 1.36). Digital skills were lower in those with eyesight problems in all domains. Digital skills did not differ significantly between those with and without hand problems (flexion deformity) (Supplementary Table S1). 

**Figure 2:**
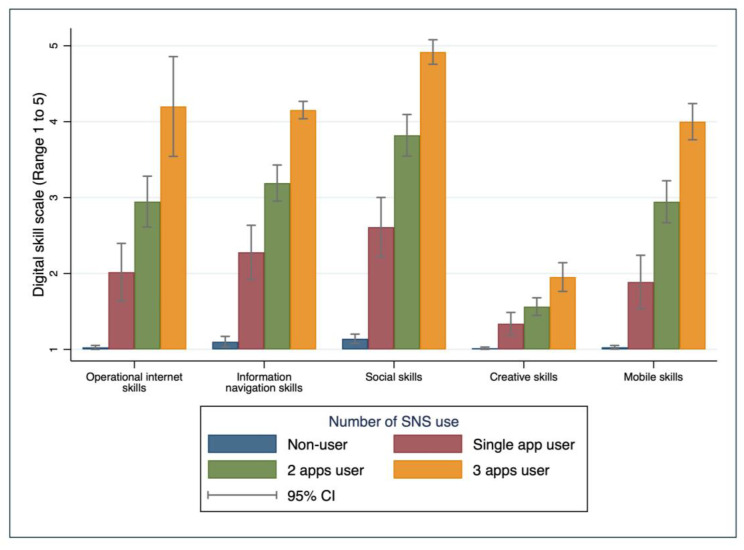
Distribution of five types of digital skills across number of SNS use among community older adults in the Republic of Korea (N = 434).

In relation to internet usage characteristics, ‘operational skills’, ‘information-navigation skills’, ‘social skills’, ‘creative skills’, and ‘mobile skills’ mean scores were higher amongst those who had access to the internet (Supplementary Tables S1 and S2). Compared to internet non-users, mobile internet users and broadband users showed a higher level of digital skills in all five skill domains. Owning a digital device corresponded to a higher level of digital skills (Supplementary Table S1). Digital skills in all domains increased as time spent on the internet increased. However, ‘operational’, ‘social’, and ‘creative’ skills were observed to be higher among ‘regular users’ (4 to 24 h per week on the internet) compared to ‘broad users’ (more than 24 h per week). Compared to ‘non-users’, all types of ‘SNS users’ had higher digital skills. Among the different types of SNS, ‘Facebook’ users had the highest digital skills, especially ‘social skills’ (4.94 ± 0.29). The number of SNS applications used showed significant differences between the groups. Older adults who used three SNS applications scored the highest digital skill across all five domains (Figure 2).

#### 3.1.3. Association of Digital Skills with Self-Rated Health and Digital Technology Use for Health Promotion Activities

In relation to perceived health status, 65 (14.98%) of the study participants reported that they were ‘very healthy’; 127 (29.26%) were ‘moderately healthy’; 150 (34.56%) were ‘not very healthy’; and 92 (21.20%) were ‘not healthy’. The responses for participation in health promotion activities revealed varying frequencies of behaviors among the participants. The majority (81.52%) indicated that they ‘never’ use the internet and digital technology to improve eating habits. Smaller percentages reported engaging in this behavior- ‘rarely’ (8.08%), ‘sometimes’ (4.16%), ‘usually’ (3.23), and ‘often’ (3%). Internet and technology use to access healthcare closely mirrored the above item, with 80.65% reporting that they ‘never’ engaged in this activity and 8.06% ‘rarely’ doing so. The responses for ‘sometimes’, ‘often’, and ‘usually’ engaging in this behavior were low, with percentages ranging from 3% to 4%. The usage of the internet and digital technology to access care services for older persons showed lower usage, with 85.45% answering that they ‘never’ engaged in this behavior. A minority of the participants reported occasional engagement in this behavior, with 8.78% choosing ‘rarely’, and 1–2% reporting ‘often’ or ’usually’ (Table 3).

**Figure 3:**
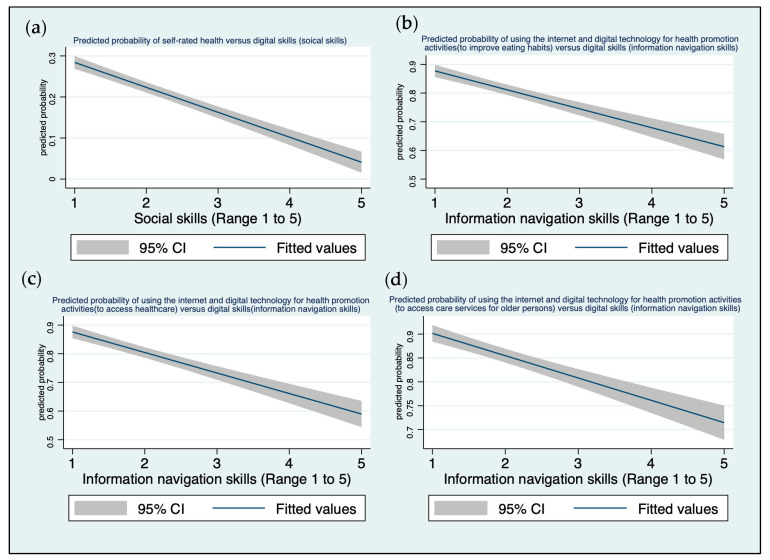
Relationship between digital skills and health-related outcomes: (**a**) predicted probability of self-rated health (unhealthy) and digital skills (social skills); (**b**) predicted probability of ‘Never’ using the internet and digital technology for health promotion activities (to improve eating habits) and digital skills (information navigation skills); (**c**) predicted probability of ‘Never’ using the internet and digital technology for health promotion activities (to access healthcare) and digital skills (information navigation skills); (**d**) predicted probability of ‘Never’ using the internet and digital technology for health promotion activities (to access care services for older persons) and digital skills (information navigation skills).

Multivariable ordinal logistic regression analysis of participants showed that ‘social skills’ was a significant predictor of self-rated health status when demographic variables were controlled (gender, age, education level, and income) (β = 0.37, 95%CI = 0.08, 0.65, *p*-value < 0.05) (Table 4) (Figure 3a). For a one unit increase in ‘information-navigation skills’, we expect a 0.43 increase in the log odds of being in a higher level of using the internet and digital technology to improve eating habits (β = 0.43, 95%CI = 0.09, 0.77, *p*-value < 0.05) (Table 5) (Figure 3b). Similarly, ‘information-navigation skills’ was positively associated with internet and digital technology use to access healthcare (β = 0.53, 95%CI = 0.21, 0.85, *p*-value < 0.01) (Table 6) (Figure 3c) and to access care services for older persons (β = 0.45, 95%CI = 0.11, 0.79, *p*-value < 0.01) (Table 7) (Figure 3d).

### 3.2. Qualitative Findings

Analysis of focus group transcripts revealed participants’ emphasis on types of digital skills utilized for social connection and accessing information in a unique Korean context. Predominant themes were identified as follows: (i) social connection; (ii) information access for daily activities; and (iii) information access for health and participation in health promotion activities. Qualitative findings further supported the quantitative results (Table 8 and Table 9).

#### 3.2.1. Social Connection

Individual or group chat using Kakao Talk was mostly used by the study participants for social connection and information sharing, including health information, among family members and peer groups. Some participants connected with each other by text messaging and sharing photos. Older adults with limited digital skills could receive messages and calls. However, they did not know how to reply to messages or make return calls.

“Nowadays, all group gatherings are done through Kakao Talk”.

“I can receive calls and messages from friends and children, but I don’t know how to reply back to them”.

“We took pictures and made videos when we went on a trip. I edited them nicely and sent them to everyone, made albums and sent them as well”.

Among the digital skills, ‘social skills’ were mostly utilized, understanding who to share with, and what health information to share, supported by basic ‘operational skills’ and ‘mobile skills’ for operating smartphones and mobile phones. ’Creative skills’ were applied among the advanced users. During the interview, one participant mentioned that he created photo albums and video clips and then shared them to his peer group.

#### 3.2.2. Information Access for Daily Activities

To retrieve information, the search engine Naver, often known as the “Google of Korea”, was mostly used. Participants spoke the keywords via the AI speaker and found information such as for navigation and shopping. The digital skill type of ’information navigation’ was mostly mentioned for selecting the best keywords and navigating websites.

“For example, when I go to Cheongnyangni or somewhere, I use Naver or Daum. I use Naver a lot. I type ’Cheongnyangni’ and check the route”.

“I ask here (Google Voice) and ask for all the side dishes”.

“If there is no cakes size that I want near my place, I buy it from different neighborhood just like I searched Paris Baguette here”.

#### 3.2.3. Information Access for Health and Participation in Health Promotion Activities

Individual participants found health information via YouTube and Naver for exercise, and to relieve certain symptoms. Some shared health information through Kakao Talk, forwarding messages to family, peers, and groups. One particular health application was mentioned linking physical activities (ex. walking), and recreation (ex. planting trees), with reward incentives in the real world (ex. actual plants). The utilization of the ‘information navigation’ digital skill, combined with that of ‘social skills’ were mostly observed. Telehealth programs were provided at the primary healthcare centers. Education programs for older adults, especially digital literacy trainings were available at these centers to enhance digital skills among older persons. However, participants needed to own a mobile phone and have access to the internet to participate in the programs. 

“YouTube! I can search some of the information to release pain”.

“That’s high blood pressure. Sometimes, when I get an entire Kakao Talk message like this, it comes from there. And if you click on it, there is a lot of information. People send it to each other like this. But it’s all right there, even Naver. So, it becomes information and the deeper you go, the more you see this and that”.

“There is an app called Corestep, and if you step a certain distance, plants grow, and if you take good care of the plants, they actually send you real plants”.

“I’ve searched on the internet for information about the efficacy and functions of various things. I mostly use Naver Nowadays, I use ‘AskUp’. For example, I looked for if the gardenia fruit is good for insomnia because someone said it was. I also searched for burdock tea, which has many benefits and is good for women too”.

Thematic analysis revealed that the older adults used the internet through local resources in the country context in the native Korean language, with the aid of voice commands. Through the participants’ narratives, we understood that amongst the different types of digital skills, ‘social skills’ and ‘information navigation skills’ were the most commonly applied for social connection and retrieving health information, which was consistent with our quantitative findings (Table 8 and Table 9). 

## 4. Discussion

The Republic of Korea, an aged society, is becoming increasingly digitalized in terms of health promotion and care services. As universal access to the internet and broadband has been implemented throughout the country, it is timely to explore the extent of internet use and the competency of digital skills among older persons, and its association with health-related outcomes. The present mixed-method study identified five types of digital skills: ‘operational internet skills’, ‘information navigation skills’, ‘social skills’, ‘creative skills’, and ‘mobile skills’ of Korean older adults, their internet usage characteristics, and the influence of digital skills on their self-rated health and participation in health promotion activities. Integrating quantitative and qualitative findings provided the opportunity to understand the type of digital skills that needed to be strengthened and the mechanism to encourage internet and digital technology use for healthy aging. 

The study participants excelled in ‘social skills’ above all other types of digital skill (2.16 ± 1.61), followed by ’information navigation skills’, ‘operational skills’, ‘mobile skills’, and ’creative skills’ (1.23 ± 0.48) (Table 2). To the best of our knowledge, this study is the first to measure different types of digital skills among the older population in Asia. Previous studies conducted within the general populations of the Netherlands, Italy, and Slovenia revealed that ‘operational internet skills’ scored the highest, closely followed by ‘social skills’ [30,42,43]. The variation in findings compared to earlier research might indicate differing perceptions of internet use among older adults in different contexts. Results from qualitative analysis showed the application of ‘social skills’ more frequently through chatting and sharing information via social media services (Table 8 and Table 9). ‘Creative skills’ are the most challenging among the five types of digital skills, since they test the ability to create and share quality contents that are published publicly. Our study is consistent with previous studies in finding that ‘creative skills’ were the least developed among the study participants since the majority of them were not professionals in digital technology. Findings from the focus groups revealed that the older adults applied basic digital skills, mainly ‘social skills’ and ‘information navigation skills’, with limited ‘operational skills’ and ‘mobile skills’ for daily activities such as receiving messages, calls, and sending photos on their smart phones. 

Digital skills significantly differed across socio-demographic backgrounds. The female gender, older-age groups, and those with lower education status, lower income, and eyesight problems, reported lower levels of digital skills in all five skill domains (Supplementary Table S1). Previous studies showed that education and income levels are the most influential factors in the accessibility and use of digital devices [44]. Older adults and people from disadvantaged socioeconomic groups are in the greatest need of health information and care since they generally have more health problems. However, these individuals use the internet and digital technology less [36,45,46]. They represented the majority within the categories in our study sample: female (72.58%), >75 years old (59.72%), no current income (62.21%), junior high school graduate and lower (66.13%), and those with eyesight problems (65.59%) (Table 1). Therefore, it is important to ensure that these individuals with diverse backgrounds are equipped with a sufficient level of digital skills to enable them to obtain maximum benefit from the internet to promote their health and well-being.

The current study also identified the internet usage characteristics of the study participants. Approximately half of the older adults in our sample use a digital device(s) (49.77%) and use mobile internet or broadband internet at home (45.03%) (Table 2). According to the annual survey conducted by the Korean Ministry of Science and ICT in 2022, 76.5% used smartphones, while 76.6% of older adults used the internet [20], representing a relatively higher usage than in the present study. Geographical differences in the data collection sites, which were in rural regions, might explain this lower internet penetration and digital technology usage [47]. A previous study in Hong Kong similarly found that older adults remain a disadvantaged group even in a society that has a well-developed internet infrastructure and a high internet penetration rate [48]. Regarding Social Networking Service (SNS) usage, approximately two in five of the study participants use SNS (42.40%). The most frequently used services were Kakao Talk (37.79%), YouTube (29.03%), and Facebook (5.30%) (Table 2). A recent study among older adult internet users in South Korea found that 90–95% receive or send messages by Telegram, Kakao Talk or others, and 38% use Facebook, Twitter, or others [49]. Since the current study intended to include both internet users and non-users, it was able to identify the first-degree digital gap and second-degree digital gap among the older adults of a rural area of South Korea. We also quantified internet usage hours per week in this study. One-third of the participants were regular users of the internet who spent 4 to 24 h per week using it (Table 2). The median time that internet users spent online per week was 7 (5–14) hours, which was relatively lower than that in the national survey which showed an average internet usage of 13.2 h per week for those aged 60 and older [20]. Being a relatively older cohort in our sample might explain this lower internet and digital technology usage. The mean age in our sample was 76.75 ± 6.55 years, whilst the old-old groups (75 to 84 years old) accounted for 43.98% (Table 1). It was found that internet use decreases with increasing age. The large proportion of internet non-users in our sample might have affected the overall amount of internet use and the level of digital skills. Hence, our study has been able to shed light on the real-life situation of older adults in a rural area who require empowerment and inclusion in the digital society.

**Figure 4:**
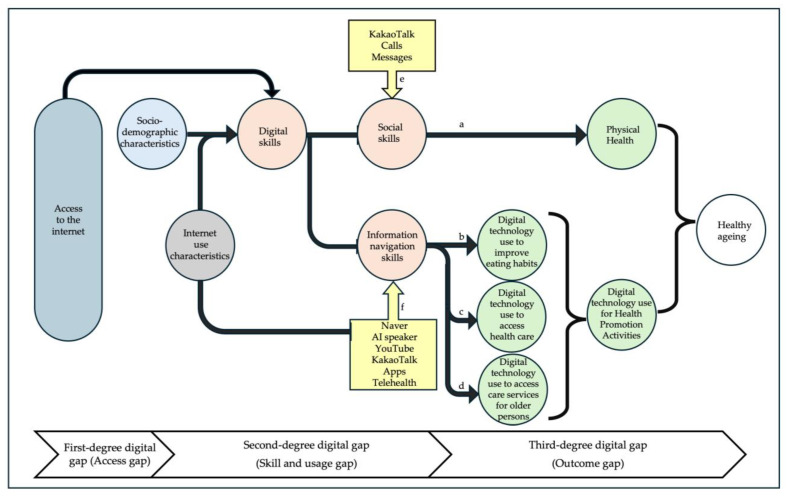
Conceptual framework constructed with the summary of the findings for digitally inclusive, healthy aging communities (DIHAC) in the Republic of Korea context; a (β = 0.36, 95%CI = 0.07, 0.65, *p*-value < 0.05); b (β = 0.43, 95%CI = 0.09, 0.77, *p*-value < 0.05), c (β = 0.53, 95%CI = 0.21, 0.85, *p*-value < 0.05), d (β = 0.45, 95%CI = 0.11, 0.79, *p*-value < 0.01), e, f qualitative findings.

The study identified the level of digital inclusion of older adults by investigating various types of digital skills and their consequences to health outcomes. One of these skills, ‘social skills’ had a significant association with self-rated health status (β = 0.36, 95%CI = 0.07, 0.65, *p*-value < 0.05) (Table 4) (Figure 3a). ‘Social skills’ or ‘communication skills’ are progressively important in the network society for social connection and exchange of information [36]. This finding is consistent with a recent study among older adults in South Korea, which found that the purposes of internet use were significantly associated with depressive symptoms and self-rated health [49]. The current study also explored how older adults applied ‘social skills’ in their daily lives (Table 8 and Table 9) (Figure 4). In Korea, Kakao Talk is the most frequently used messaging app across the country, which is consistent with our finding that the most used SNS in our study sample was Kakao Talk (37.79%). The focus group findings also revealed that the older adults could stay connected and keep sharing information and media with their friends through online apps, although there were individual differences in digital skill levels. Online social connection leads to better health by combating loneliness, social isolation, and depression [50]. Moreover, our study showed that the older adults were more confident with ‘social skills’ than all other digital skills (Table 2). If ‘social skills’ can be strengthened among older adults, their social network will become more active through SNS, and consequently, this may lead to healthy aging (Figure 4).

When older people have higher levels of digital skills, such as ‘information navigation skills’, they can modify their lifestyle to be healthier (Table 5, Table 6 and Table 7) (Figure 3). We found that usage of digital technology and the internet for a healthy lifestyle was positively associated with ‘information navigation skills’ for eating habits (β = 0.43, 95%CI = 0.09, 0.77, *p*-value < 0.05), to access healthcare (β = 0.53, 95%CI = 0.21, 0.85, *p*-value < 0.05), and to access long-term care services for older persons (β = 0.45, 95%CI = 0.11, 0.79, *p*-value < 0.01). As it is crucial to evaluate information from search engines and other online sources, ‘information navigation skills’ are the most important of all five domains of digital skills [36]. Furthermore, individuals with higher ‘information-navigation skills’ could adequately retrieve better health information than those with poor digital skills, reducing the risks of believing disinformation or fake news. The qualitative findings also revealed that the search engine Naver in native Korean language was most commonly used by Korean older adults to improve eating habits. Advanced internet users were able to find nutrition information via the AI chat box. Furthermore, ‘Information navigation skills’ facilitate better access to healthcare and long-term care services for older adults through telehealth programs, sharing health information via Kakao Talk, utilizing platforms such as YouTube and Naver and using apps for physical activity (Table 8 and Table 9). 

The qualitative findings also revealed that digital skills, especially using a mobile phone, are indispensable for the older population to access telehealth. In one of the telehealth programs mentioned in the focus group, monitoring devices were connected to the participants’ smartphones, and data were sent to the app. Consultations with healthcare professionals were virtually held. Additionally, information was shared via Kakao Talk among peer groups. Therefore, having access to the internet and a mobile phone, as well as a basic level of ‘information navigation skills’ are required to participate in health promotion programs and obtain the full benefits for their health. However, four in five participants never used the internet and digital technology for health promotion activities (Table 3). Since health information and services are transforming to be online, potentially becoming exclusively online in the future, empowering older people to be able to use online health services and leveraging ‘information-navigation skills’ are vital for the digital inclusion of this older population.

In the qualitative interviews, the study participants applied five types of digital skills for healthy and active aging. The previously mentioned ‘social skills’ and ‘information navigation skills’ have a direct association with self-rated health and participation in health promotion activities. These skills were supported by ‘operational internet skills’ and ‘mobile skills’ (Table 8 and Table 9). Button knowledge, such as being able to bookmark a website or install apps on mobile phones is the basis towards further steps in using, for example, Kakao Talk for social connection, Naver for navigation, or YouTube for health-related information. ‘Creative skills’ were developed in advanced internet users by creating new video clips and photo collages, which were then sent to peer groups. These activities indirectly contribute to the health and well-being of older adults. In the quantitative phase of the study, the multivariable analysis did not find statistically significant association of health-related outcomes with operational internet skills, creative skills, or mobile skills; although in univariate analysis, positive associations were found between self-rated health, and participation in health promotion activities. All five types of digital skills are interrelated. Therefore, boosting the ‘social skills’ and ‘information navigation skills’ among the older adults is believed to leverage ‘operational skills’, ‘mobile skills’, and potentially ‘creative skills’ as well. 

In addition to boosting individual digital skills among the older population, creating supportive environments is one of the key action areas for health promotion and health equity. In recent years, the Korean government has been focusing on digitalization in the healthcare and long-term care sectors, with initiatives carried out by the Korea Health Promotion Institute (KHPI), such as the “AI-IoT-based healthcare project for senior citizens” [51]. In this program, wearable devices and digital monitoring instruments are provided at a flat rate. However, only older adults who own a smartphone can participate in the program. Older adults who do not have physical access to the internet are excluded, further reinforcing social inequality. The qualitative findings in the present study revealed that the older adults with smartphones had better digital skills to adopt healthy behaviors such as using health apps, YouTube, and the search engine Naver for physical activities or exchanging information through Kakao Talk individually or via group chat. It is anticipated that digital technology will play a crucial role in reorienting healthcare and long-term care services. Therefore, having a basic level of digital skills is necessary for every older adult to access those services for health promotion. Healthy behaviors can be maximized when environments and policies support healthy choices, and individuals are motivated and educated to make those choices [52]. Therefore, it is important to leverage digital skills among older persons, especially regarding ‘information navigation skills’ and ‘social skills’, enabling them to operate a digital device to gain better healthcare access, make informed choices for lifestyle modification, and bring about social inclusion. 

## 5. Conclusions

The current study described the levels of five types of digital skills in older persons and the specific type of digital skills associated with self-rated health and participation in health promotion activities. The evidence in this study conclusively stated that socio-demographic characteristics contribute to the skill gap and usage gap (second-degree digital gap), which consequently results in inequality in health outcomes (third-degree digital gap) (Figure 4). ‘Social skills’ and ‘information navigation skills’ are associated with health through participation in health promotion and lifestyle modification. Digital skills are therefore fundamental to their activities of daily life in relation to social inclusion and healthy aging. As health promotion and care services are increasingly digitalized, ensuring initial and sustained use of the internet and leveraging digital skills among Korean older adults to promote a healthy lifestyle is imperative. Digital inclusion is expected to enhance their autonomy, social connection, and healthy aging, consequently leading to digitally inclusive healthy aging communities (DIHAC).

### 5.1. Strength and Limitations

Digital skill measurement of community resident older adults, in a rural area of the Republic of Korea, as conducted in this study, is new to the literature. We quantified the internet use as hours per week and digital skills as measured scores using validated instruments. This led to more accurate regression models. Another strength of the study is the application of an explanatory sequential mixed-method approach in which the qualitative findings helped to explain the quantitative findings. This study offered a more comprehensive understanding of the gray digital divide as well as digital inclusion among older persons in a city-level community-based sample. It examined older adults’ digital skills and their ability to utilize technology, and in so doing, revealed the remaining digital gaps. This study is not without limitations. The nature of the cross-sectional design may limit the associations to be interpreted as causal relations. In addition, we did not use any kind of equipment such as pedometers to measure actual health behavior. In the future study, more specific behavior measurements can be applied to measure health behaviors using digital technology such as pedometers, body composition, wearable devices, or health apps. Longitudinal follow up may further confirm the associations. Furthermore, since the study included both internet users and non-users, it might underestimate the digital skills level of the sample overall.

### 5.2. Implication of the Study

As digital technology has become a necessity for the daily activities of older Koreans, those without sufficient digital skills are at risk of being left behind. The digital divide among older adults still remains especially among socially disadvantaged groups such as the very old, women, and those with impoverished backgrounds. This study identified the diversity of internet access, digital technology use, digital skills, and their interrelatedness with health consequences. Older adults face several challenges in developing their digital skills. Firstly, rapid advances in digital technology leads to less willingness to develop the digital skills necessary to adapt to these new software interfaces and devices. Secondly, memory and cognitive decline, associated with aging, hinders older adults’ ability to remember new information. Furthermore, older people may struggle with blurred vision and hand tremors when operating digital devices. Older adults may experience fear and stress about phishing scams or making mistakes that can cause irreversible damage to devices. Additionally, the lack of tailored support and digital literacy training programs in the community for older adults can hinder their ability to maintain and update their digital skills over time. Another overlooked challenge for older adults is the potential impatient response from their children and grandchildren shown during the teaching process, who are typically more familiar with digital technologies. This lack of patience can lead to a lower sense of self-efficacy among older adults, making them less likely to engage in learning digital skills.

The second-degree digital gap among the older adults indicates that it is imperative for older people to develop or upskill their levels of digital skills in order to obtain the full benefits of the internet through education programs and support systems tailored to their needs. Empowering older adults with digital inclusion should be considered at different levels across multiple sectors. At the macro-level, policies and education programs for digital literacy training for older adults with synergistic actions between government, NGOs, and the private sector, such as internet service providers (ISPs), will maximize the opportunities for healthy aging. Training older people how to use various apps, including SNS for social connection, sharing information, and daily activities, and health apps for monitoring of health and well-being, as well as training on operating KIOSKs, and safety measures to avoid phishing scams, will level up their digital skills and promote their health and well-being. In addition, organizations are recommended to assess their policy and operations, ensuring information and services are accessible to older adults in an age-friendly approach, especially when services are rapidly transformed to be exclusively online. Improvement in the recruitment policy to digital literacy programs for older adults, especially to those with low willingness to adopt digital technologies, should be considered to increase the participation rate [53].

Education programs for the older people such as digital literacy trainings can also be implemented at the meso-level by local municipalities at community or welfare centers with local resources, either as new community-based social innovations (CBSIs) [54] or integrating digital technologies into existing activities. For example, teaching older people in community-developed physical exercise, social, or cultural programs to use SNS or apps that can reskill or upskill their digital skills will lead to social inclusion, better information access, and connection between peer groups or administrators. Strengthening community actions with inter-generational digital education programs or peer training strategies, such as volunteerism, has the potential to increase inclusivity and sustained productivity. At the micro-level, empowering family members, such as the spouse, children, and grandchildren, or the peer groups of older adults, creates a supportive environment and will promote digital technology use among older adults, and with it, active and healthy aging. In the post-COVID-19 pandemic era, ensuring digital inclusion for aging communities has never been more crucial for their well-being. Our study’s implications extend to culturally similar countries facing a gray digital divide with an increasing aging population. By leveraging digital skills among older people across different levels, they will be able to stay informed, connected, and engaged in health promotion activities, ultimately leading to healthy aging.

## Figures and Tables

**Table 1:** Socio-demographic characteristics of participants in the survey (N = 434).

Variables		Frequency (Percentage)	Mean ± SD
Gender	Male	119 (27.42)	
	Female	315 (72.58)	
Age (Years)	Young-old (65–74 years)	174 (40.28)	
	Old-old (75–84 years)	190 (43.98)	
	Oldest-old (aged 85 and older)	68 (15.74)	
			76.75 ± 6.55
Education status	Did not go to school	70 (16.13)	
	Primary school graduate	124 (28.57)	
	Junior high school graduate	93 (21.43)	
	High school graduate	102 (23.50)	
	University graduate or higher	45 (10.37)	
Income	No current income	270 (62.21)	
	≤KRW 1 million	71 (16.36)	
	KRW 1–2.5 million	65 (14.98)	
	≥KRW 2.5 million	28 (6.45)	
Pension	Yes	385 (88.71)	
	No	49 (11.29)	
Eye problems in using a digital device	Yes	265 (65.59)	
	No	139 (34.41)	
Hand problems in using a digital device (example: flexion deformity)	Yes	9 (2.23)	
	No	395 (97.77)	

**Table 2:** Digital skills and internet usage characteristics of participants in the survey (N = 434).

Variables		Frequency (Percentage)	Mean ± SD
Digital skills (Range: 1 to 5)	Operational internet skills		1.77 ± 1.44
	Information navigation skills		1.90 ± 1.36
	Social/communication skills		2.16 ± 1.61
	Creative skills		1.23 ± 0.48
	Mobile skills		1.74 ± 1.29
Access to the internet	Having access No access	191 (44.01) 243 (55.99)	
Types of home internet environment ^a^	Non-user	238 (54.97)	
	Mobile internet	81 (18.71)	
	Broadband (Fiber, ADSL)	136 (31.41)	
	Do not know	2 (0.46)	
Types of digital devices use ^a^	Non-user	218 (50.23)	
	Smartphone	206 (47.47)	
	Mobile phone	8 (1.84)	
	Personal computer	42 (9.68)	
	Tablet	3 (0.69)	
Digital device ownership	Non-user	218 (50.23)	
	Single device owner	176 (40.55)	
	2 devices owner	37 (8.53)	
	3 devices owner	3 (0.69)	
Time spent on the internet (hours per week)	Non-user (0)	227(56.19)	
	Low users of the Internet (<4)	33 (8.17)	
	Regular users (4 to 24)	134 (33.17)	
	Broad users (>24)	10 (2.48)	
Types of SNS use ^a^	Non-user	250 (57.60)	
	Kakao Talk	164 (37.79)	
	YouTube	126 (29.03)	
	Facebook	23 (5.30)	
	Line	12 (2.76)	
	Facebook Messenger	1 (0.23)	
	Others (Band)	1 (0.23)	
SNS use by number of groups	Non-user	250 (57.60)	
	Single app user	58 (13.36)	
	2 apps user	109 (25.12)	
	3 apps user	17 (3.92)	

Note: ^a^ Data on types of home internet environment, types of digital device use, and types of SNS use were mutually non-exclusive. Only the number and percentage of those answering “Yes” were described to improve readability.

**Table 3:** Self-rated health and participation in health promotion activities among community resident older adults in the Republic of Korea (N = 434).

Variables		Frequency (Percentage)
Self-rated health		
Self-rated health status	Very healthy	65 (14.98)
	Moderately healthy	127 (29.26)
	Not very healthy	150 (34.56)
	Not healthy	21.20)
Participating in Health Promotion Activities		
Usage of the internet and digital technology to improve eating habits	Never	353 (81.52)
	Rarely	35 (8.08)
	Sometimes	18 (4.16)
	Often	13 (3.00)
	Usually	14 (3.23)
Usage of the internet and digital technology to access healthcare	Never	350 (80.65)
	Rarely	35 (8.06)
	Sometimes	20 (4.61)
	Often	15 (3.46)
	Usually	14 (3.23)
Usage of the internet and digital technology to access care services for older persons (e.g., house assistance, house cleaning, home bathing, and others)	Never	370 (85.45)
	Rarely	38 (8.78)
	Sometimes	15 (3.46)
	Often	4 (0.92)
	Usually	6 (1.39)

**Table 4:** Association of digital skills with self-rated health among community resident older adults in the Republic of Korea (N = 432).

Variable		Univariate Analysis			Multivariable Analysis		
		β	95% CI	*p*-Value	β	95% CI	*p*-Value
Digital skills							
	Operational skills	0.44 *	0.32–0.57	<0.001	−0.07	−0.34–0.20	0.600
	Information navigation skills	0.48 *	0.35–0.62	<0.001	−0.02	−0.32–0.27	0.874
	Social skills	0.47 *	0.35–0.59	<0.001	0.37 *	0.08–0.65	0.013
	Creative skills	1.38 *	0.98–1.78	<0.001	0.51	−0.11–1.14	0.108
	Mobile skills	0.47 *	0.32–0.61	<0.001	−0.23	−0.57–0.11	0.189
Income							
	No current income	0 (Ref)			0 (Ref)		
	≤KRW 1 million	0.71 *	0.25–1.17	0.002	1.05 *	0.53–1.57	<0.001
	KRW 1–2.5 million	0.23	−0.27–0.73	0.372	−0.27	−0.83–0.29	0.351
	≥KRW 2.5million	0.34	−0.35–1.03	0.335	0.25	−0.53–1.03	0.527
Education							
	Did not go to school	0 (Ref)			0 (Ref)		
	Primary school graduate	0.91 *	0.35–1.47	0.002	0.61 *	0.02–1.21	0.044
	Junior high school graduate	0.98 *	0.38–1.58	0.001	0.72 *	0.09–1.35	0.025
	High school graduate	2.05 *	1.44–2.65	<0.001	1.40 *	0.71–2.08	<0.001
	University graduate or higher	2.46 *	1.73–3.19	<0.001	1.40 *	0.49–2.30	0.002
Age		−0.13 *	−0.16–−0.10	<0.001	−0.07 *	−0.11–−0.03	<0.001
Gender							
	Male	0 (Ref)			0 (Ref)		
	Female	0.03	−0.35–0.41	0.870	0.19	−0.25–0.62	0.400

Note: Self-rated health status was measured by 4-point Likert scale ranging from 1 to 4: very healthy to not healthy. * *p*-value < 0.05, β = ordinal logistic regression, regression coefficient.

**Table 5:** Association of digital skills with internet and digital technology use for health promotion activities to improve eating habits among community resident older adults in the Republic of Korea (N = 431).

Variable		Univariate Analysis			Multivariable Analysis		
		β	95% CI	*p*-Value	β	95% CI	*p*-Value
Digital skills							
	Operational skills	0.18 *	0.03–0.34	0.020	−0.07	−0.45–0.32	0.741
	Information navigation skills	0.39 *	0.23–0.55	<0.001	0.43 *	0.09–0.77	0.013
	Social skills	0.17 *	0.03–0.31	0.020	−0.15	−0.58–0.28	0.492
	Creative skills	0.71 *	0.27–1.16	0.002	0.28	−0.55–1.11	0.513
	Mobile skills	0.17	0.00–0.35	0.053	0.04	−0.42–0.51	0.854
Income							
	No current income	0 (Ref)			0 (Ref)		
	≤KRW 1 million	2.46 *	1.84–3.09	<0.001	2.82 *	2.10–3.55	<0.001
	KRW 1–2.5 million	0.97 *	0.18–1.77	0.016	1.02 *	0.15–1.90	0.022
	≥KRW 2.5 million	2.35 *	1.46–3.25	<0.001	3.03 *	1.96–4.10	<0.001
Education							
	Did not go to school	0 (Ref)			0 (Ref)		
	Primary school graduate	−0.37	−1.13–0.39	0.342	−0.07	−1.01–0.86	0.877
	Junior high school graduate	−0.45	−1.29–0.38	0.288	0.39	−0.66–1.44	0.462
	High school graduate	0.21	−0.53–0.95	0.574	1.12 *	0.06–2.19	0.038
	University graduate or higher	0.67	−0.20–1.53	0.131	1.21	−0.10–2.52	0.071
Age		−0.03	−0.06–0.01	0.177	0.02	−0.04–0.07	0.590
Gender							
	Male	0 (Ref)			0 (Ref)		
	Female	0.64 *	0.02–1.26	0.043	1.39 *	0.62–2.17	<0.001

Note: Digital technology use for health promotion activities to improve eating habits was measured by 5-point Likert scale ranging from 0 to 4; Never to Usually. * *p*-value < 0.05, β = ordinal logistic regression, regression coefficient.

**Table 6:** Association of digital skills with internet and digital technology use for health promotion activities to access healthcare among community resident older adults in the Republic of Korea (N = 432).

Variable		Univariate Analysis			Multivariable Analysis		
		β	95% CI	*p*-Value	β	95% CI	*p*-Value
Digital skills							
	Operational skills	0.19 *	0.04–0.34	0.014	−0.11	−0.48–0.26	0.573
	Information navigation skills	0.43 *	0.27–0.59	<0.001	0.53 *	0.21–0.85	0.001
	Social skills	0.18 *	0.04–0.32	0.013	−0.21	−0.64–0.22	0.336
	Creative skills	0.72 *	0.28–1.16	0.001	0.15	−0.68–0.98	0.726
	Mobile skills	0.20 *	0.03–0.37	0.023	0.13	−0.33–0.59	0.579
Income							
	No current income	0 (Ref)			0 (Ref)		
	≤KRW 1 million	2.56 *	1.94–3.17	<0.001	2.96 *	2.24–3.69	<0.001
	KRW 1–2.5 million	1.11 *	0.33–1.88	0.005	1.24 *	0.38–2.11	0.005
	≥KRW 2.5 million	2.38 *	1.49–3.28	<0.001	3.00 *	1.94–4.05	<0.001
Education							
	Did not go to school				0 (Ref)		
	Primary school graduate	−0.28	−1.03–0.48	0.473	0.32	−0.63–1.26	0.515
	Junior high school graduate	−0.32	−1.14–0.50	0.448	0.86	−0.20–1.92	0.113
	High school graduate	0.32	−0.42–1.05	0.396	1.59 *	0.54–2.65	0.003
	University graduate or higher	0.85	0.00–1.71	0.051	1.75 *	0.46–3.05	0.008
Age		−0.02	−0.06–0.01	0.205	0.03	−0.03–0.09	0.279
Gender							
	Male	0 (Ref)			0 (Ref)		
	Female	0.63 *	0.03–1.23	0.041	1.37 *	0.61–2.12	<0.001

Note: Digital technology use for health promotion activities to access healthcare was measured by 5-point Likert scale ranging from 0 to 4; Never to Usually. * *p*-value < 0.05, β = ordinal logistic regression, regression coefficient.

**Table 7:** Association of digital skills with internet and digital technology use for health promotion activities to access care services for older persons among community resident older adults in the Republic of Korea (N = 431).

Variable		Univariate Analysis			Multivariable Analysis		
		β	95% CI	*p*-Value	β	95% CI	*p*-Value
Digital skills							
	Operational skills	0.14	−0.03–0.31	0.101	0.04	−0.39–0.48	0.843
	Information navigation skills	0.36 *	0.18–0.54	<0.001	0.45 *	0.11–0.79	0.009
	Social skills	0.12	−0.04–0.27	0.152	−0.08	−0.54–0.37	0.721
	Creative skills	0.57 *	0.09–1.06	0.021	0.29	−0.64–1.21	0.540
	Mobile skills	0.08	−0.12–0.28	0.420	−0.27	−0.76–0.23	0.293
Income							
	No current income	0 (Ref)			0 (Ref)		
	≤KRW 1 million	2.55 *	1.84–3.27	<0.001	2.45 *	1.68–3.22	<0.001
	KRW 1–2.5 million	1.29 *	0.42–2.17	0.004	1.39 *	0.46–2.33	0.003
	≥KRW 2.5 million	2.36 *	1.39–3.33	<0.001	2.59 *	1.45–3.72	<0.001
Education							
	Did not go to school	0 (Ref)			0 (Ref)		
	Primary school graduate	−0.30	−1.07–0.48	0.457	0.13	−0.81–1.07	0.787
	Junior high school graduate	−0.97	−1.94–0.01	0.052	−0.35	−1.52–0.82	0.556
	High school graduate	−0.25	−1.06–0.56	0.552	0.33	−0.77–1.44	0.554
	University graduate or higher	0.34	−0.59–1.26	0.475	0.63	−0.75–2.02	0.370
Age		−0.01	−0.05–0.03	0.602	0.02	−0.04–0.08	0.519
Gender							
	Male	0 (Ref)			0 (Ref)		
	Female	0.42	−0.23–1.07	0.209	0.86 *	0.08–1.64	0.031

Note: Digital technology use for health promotion activities to access care services for older persons was measured by 5-point Likert scale ranging from 0 to 4; Never to Usually. * *p*-value < 0.05, β = ordinal logistic regression, regression coefficient.

**Table 8:** Thematic framework in qualitative analysis with example codes emerged from focus group interviews of community resident older adults in South Korea.

Interview Transcripts	Description In-Vivo Codes	Tentative Theme	Core Concept
“These activities (education on using digital health equipment, mobile phone, AI speaker training) helped the older adults to be more socially connected and sharing information with each other, making friends, and working together by using digital technology” (Female participants)	“Share information” “Socially connected” “Making friends” “Using digital technology”	Social skills: knowing who and what to share	Social skills to share information with peer groups
“Naver homepage” (Female participant) Some respondents: “YouTube” “I can search some of the information to release pain. “I use the Naver home page and do some exercise” (Male participant)	“Naver” “YouTube” “To release pain” “Do some exercise”	Being able to find the right keywords and places to find information	Information-navigation to assess healthcare
“I can freely use text messages. I can take pictures and such” “I also use Kakao Talk a lot. Every morning, I get three messages from my friends. Then, I tell and send them” (Female participant)	“Use text messages” “Take pictures” “Kakao Talk” “Tell and send them”	Acquired basic skills to operate a digital device	Operational skills to function digital device, which facilitates social skills for social connection.
“I search through Kakao Talk to see what is good and what is bad for my health” (Female participants) “If other people send me this, I think it’s good information, so I send it to other people and forward it to them through Kakao Talk’ (Female participants”	“Search through Kakao Talk” “For my health” “Good information” “Forward it to them”	Use mobile phone SNS app Mobile skills	Mobile skills to access health integrate with social skills to share information
“I can receive calls and messages from friends and children, but I don’t know how to reply back to them”	“Can receive calls and messages”	Use SNS calls and messages for social connection and receive information only	Limited operational skills and mobile skills to make social connection more effectively
“We took pictures and made videos when we went on a trip. I edited them nicely and sent them to everyone, made albums and sent them as well.”	“Took pictures” “Made videos” “Edited them” “Sent them to everyone”	Know how to create new content from existing photo and videos Feel confident to share them to others	Creative skills, social skills with basic operational internet skills, mobile skills

**Table 9:** Focus group themes and subthemes to explore usage of the internet and digital technology among community resident older adults in South Korea.

Theme	Sub-Theme	Digital Skills
Social connection	(i) SNS: Kakao Talk (a) Individual (b) Group chat (ii) Text messages (iii) Call	Social skills, occasional creative skills Supported by operational skills and mobile skills
Information access for daily activities	(i) Naver (ii) AI-speaker	Information navigation skills
Information access for health	(i) Sharing and being shared from Kakao Talk (ii) YouTube (iii) Naver (iv) Apps linking physical activity, and recreation with rewards in real life (v) Telehealth programs	Information navigation skills Integrated with social skills, supported by operational skills and mobile skills

## Data Availability

The data presented in this study are available on request from the corresponding author due to privacy reasons.

## Supplementary Materials

The following supporting information can be downloaded at https://www.mdpi.com/article/10.3390/ejihpe14080154/s1, Table S1 Distribution of Digital skills across socio-demographic characteristics and internet usage characteristics among community resident older adults in the Republic of Korea (N = 434); Table S2 Distributions of Digital skills of the participants in the survey and Cronbach’s Alphas for each domain (N = 434).
