# Supplementary material for: Digital Inclusion among Community Older Adults in the Republic of Korea: Measuring Digital Skills and Health Consequences
Source: Eur J Investig Health Psychol Educ. 2024 Aug 8;14(8):2314–36. doi: 10.3390/ejihpe14080154 (PMC11353258; doi:10.3390/ejihpe14080154)
Supplement: Supplementary file 1 [file ejihpe-14-00154-s001.zip › ejihpe-3133624-supplementary.pdf]

Supplementary Table S1. Distribution of Digital skills across socio-demographic characteristics and internet usage characteristics among community resident older adults in the Republic of Korea (N = 434).

**Variable**		**Frequency**	**Digital skill domains (Mean ± SD)**									
		**(Percentage)**	**Operational internet skills**		**Information Navigation skills**		**Social skills**		**Creative skills**		**Mobile skills**	
				***p*-Value**		***p*-Value**		***p*-Value**		***p*-Value**		***p*-Value**
Overall digital skills (Scale range: 1-5)			1.77 ± 1.44		1.90 ± 1.36		2.16 ± 1.61		1.23 ± 0.48		1.74 ± 1.29	
Digital Skill Distribution across Socio-demographic Characteristics												
Gender [b]	Male	119 (27.42)	2.17 ± 1.72	0.0052 **	2.24 ± 1.49	0.0104 *	2.66 ± 1.80	<0.001 ***	1.37 ± 0.58	<0.001 ***	2.13 ± 1.51	<0.001 ***
	Female	315 (72.58)	1.61 ± 1.29		1.78 ± 1.28		1.97 ± 1.49		1.18 ± 0.42		1.59 ± 1.17	
Age (Years) [c]	Young-old (65–74 years)	174 (40.28)	2.63 ± 1.82	<0.001 ***	2.71 ± 1.51	<0.001 ***	3.17 ± 1.76	<0.001 ***	1.46 ± 0.57	<0.001 ***	2.50 ± 1.56	<0.001 ***
	Old-old (75–84 years)	190 (43.98)	1.23 ± 0.75		1.42 ± 0.97		1.61 ± 1.16		1.10 ± 0.38		1.30 ± 0.83	
	Oldest-old (aged 85 and older)	68 (15.74)	1.07 ± 0.31		1.20 ± 0.73		1.14 ± 0.54		1.02 ± 0.13		1.05 ± 0.25	
Education status[c]	Did not go to school	70 (16.13)	1.21 ± 0.83	<0.001***	1.52 ± 1.17	<0.001***	1.46 ± 1.13	<0.001 ***	1.05 ± 0.18	<0.001 ***	1.26 ± 0.83	<0.001 ***
	Primary school graduate	124 (28.57)	1.14 ± 0.54		1.32± 0.88		1.45 ± 0.98		1.06 ± 0.26		1.19 ± 0.63	
	Junior high school graduate	93 (21.430	1.35 ± 1.08		1.65 ± 1.20		2.02 ± 1.50		1.11 ± 0.33		1.54 ± 1.18	
	High school graduate	102 (23.5)	2.36 ± 1.69		2.35 ± 1.44		2.75 ± 1.77		1.41 ± 0.57		2.16 ± 1.44	
	University graduate or higher	45 (10.37)	3.86 ± 1.51		3.62 ± 1.14		4.12 ± 1.43		1.85 ± 0.64		3.47 ± 1.37	
Income [c]	No current income	270 (62.21)	1.68 ± 1.43	0.0122 *	1.74 ± 1.28	0.0030 **	2.03 ± 1.59	<0.001 ***	1.18 ± 0.42	<0.001 ***	1.69 ± 1.30	0.0351 *
	≤KRW 1 million	71 (16.36)	1.59 ± 1.14		2.04 ± 1.43		1.95 ± 1.41		1.21 ± 0.53		1.57 ± 1.12	
	KRW 1–2.5 million	65 (14.98)	2.16 ± 1.65		2.28 ± 1.42		2.81 ± 1.74		1.41 ± 0.52		2.09 ± 1.36	
	≥KRW 2.5million	28 (6.45)	2.08 ± 1.56		2.24 ± 1.53		2.42 ± 1.62		1.38 ± 0.66		1.88 ± 1.37	
Pension [b]	Yes	385 (88.71)	1.79 ± 1.49	0.5523	1.88 ± 1.36	0.0413 *	2.18 ± 1.65	0.5496	1.23 ± 0.46	0.8496	1.76 ± 1.33	0.8093
	No	49 (11.29)	1.55 ± 1.02		2.09 ± 1.36		2.01 ± 1.26		1.27 ± 0.59		1.57 ± 0.99	
Eye problems in using a digital device [b]	Yes	265 (65.59)	1.60 ± 1.33	<0.001 ***	1.64 ± 1.20	<0.001 ***	1.90 ± 1.51	<0.001 ***	1.17 ± 0.41	<0.001 ***	1.59 ± 1.20	<0.001 ***
	No	139 (34.41)	2.15 ± 1.65		2.30 ± 1.47		2.74 ± 1.72		1.37 ± 0.57		2.11 ± 1.44	
Hand problems in using a digital device	Yes	9 (2.23)	1.44 ± 1.33	0.5482	1.36 ± 1.07	0.2486	1.60 ± 1.36	0.2441	1.07 ± 0.20	0.2605	1.44 ± 1.33	0.4147
(Example: Flexion deformity) [b]	No	395 (97.77)	1.79 ± 1.47		1.88 ± 1.34		2.20 ± 1.64		1.24 ± 0.48		1.77 ± 1.31	
Digital Skill Distribution across Internet Usage Characteristics												
Access to the internet	Have access	191 (44.01)	2.67 ± 1.77	<0.001 ***	2.83 ± 1.41	<0.001 ***	3.36 ± 1.62	<0.001 ***	1.49 ± 0.59	<0.001 ***	2.59 ± 1.50	<0.001 ***
	No access	243 (55.99)	1.05 ± 0.28		1.16 ± 0.70		1.19 ± 0.68		1.03 ± 0.19		1.06 ± 0.41	
Types of Internet environment	Non-user	238 (54.97)	1.04 ± 0.22	<0.001 ***	1.15 ± 0.68	<0.001 ***	1.18 ± 0.65	<0.001 ***	1.02 ± 0.15	<0.001 ***	1.04 ± 0.33	<0.001 ***
at home [a,b]	Mobile internet	81 (18.71)	1.81 ± 1.23	0.0391 *	2.07 ± 1.26	0.0080 **	2.47 ± 1.50	0.0209 *	1.33 ± 0.63	0.1594	1.93 ± 1.28	0.0022 **
	Fibre, ADSL	136 (31.41)	3.14 ± 1.81	<0.001 ***	3.25 ± 1.31	<0.001 ***	3.82 ± 1.47	<0.001 ***	1.61 ± 0.59	<0.001 ***	3.00 ± 1.48	<0.001 ***
	Do not know	2 (0.46)	2.10 ± 0.71	0.1310	2.60 ± 0.57	0.2264	3.00 ± 1.70	0.2723	1.30 ± 0.42	0.7611	1.50 ± 0.71	0.9360
Types of digital devices use [a,b]	Non-user	218 (50.23)	1.02 ± 0.22	<0.001 ***	1.09 ± 0.56	<0.001 ***	1.04 ± 0.27	<0.001 ***	1.01 ± 0.15	<0.001 ***	1.03 ± 0.29	<0.001 ***

	Smartphone	206 (47.47)	2.55 ± 1.75	<0.001 ***	2.76 ± 1.43	<0.001 ***	3.35 ± 1.61	<0.001 ***	1.46 ± 0.59	<0.001 ***	2.51 ± 1.51	<0.001 ***
	Mobile phone	8 (1.84)	1.70 ± 1.02	0.6714	1.55 ± 0.87	0.8111	2.03 ± 1.15	0.9320	1.43 ± 0.63	0.2652	1.44 ± 0.62	0.8520
	Personal Computer	42 (9.68)	3.62 ± 1.54	<0.001 ***	3.63 ± 1.04	<0.001 ***	4.07 ± 1.23	<0.001 ***	1.73 ± 0.61	<0.001 ***	3.31 ± 1.38	<0.001 ***
	Tablet	3 (0.69)	4.27 ± 0.70	0.0161 *	3.33 ± 0.50	0.0738	3.80 ± 0.72	0.0933	2.00 ± 0.20	0.0074 **	2.33 ± 1.15	0.3457
Digital device ownership [c]	Non-user	218 (50.23)	1.02 ± 0.22	<0.001 ***	1.09 ± 0.56	<0.001 ***	1.04 ± 0.27	<0.001 ***	1.01 ± 0.15	<0.001 ***	1.03 ± 0.29	<0.001***
	Single device owner	176 (40.55)	2.24 ± 1.67		2.52 ± 1.44		3.08 ± 1.64		1.39 ± 0.56		2.24 ± 1.45	
	2 devices owner	37 (8.53)	3.67 ± 1.55		3.63 ± 1.06		4.23 ± 1.12		1.70 ± 0.61		3.54 ± 1.28	
	3 devices owner	3 (0.69)	4.20 ± 0.80		3.53 ± 0.23		3.87 ± 0.70		2.20 ± 0.40		2.00 ± 1.00	
Time spent on internet (hours per week) [c]	Non-user (0)	227 (56.19)	1.05 ± 0.32	<0.001 ***	1.04 ± 0.29	<0.001 ***	1.12 ± 0.39	<0.001 ***	1.01 ± 0.08	<0.001 ***	1.01 ± 0.13	<0.001***
	Low users of the Internet (<4)	33 (8.17)	1.98 ± 1.46		2.30 ± 1.16		3.16 ± 1.37		1.42 ± 0.66		1.91 ± 1.26	
	Regular users (4-24)	134 (33.17)	2.91 ± 1.82		3.08 ± 1.41		3.67 ± 1.64		1.56 ± 0.57		2.91 ± 1.46	
	Broad users (>24)	10 (2.48)	2.82 ± 1.68		3.12 ± 1.41		3.46 ± 1.57		1.46 ± 0.82		3.20 ± 1.80	
Types of SNS use [a,b]	Non-user	250 (57.60)	1.03 ± 0.21	<0.001 ***	1.10 ± 0.56	<0.001 ***	1.14 ± 0.49	<0.001 ***	1.02 ± 0.12	<0.001 ***	1.03 ± 0.19	<0.001***
	Kakao Talk	164 (37.79)	2.84 ± 1.79	<0.001 ***	3.02 ± 1.36	<0.001 ***	3.65 ± 1.55	<0.001 ***	1.54 ± 0.61	<0.001 ***	2.81 ± 1.50	<0.001***
	YouTube	126 (29.03)	3.02 ± 1.76	<0.001 ***	3.28 ± 1.24	<0.001 ***	3.84 ± 1.45	<0.001 ***	1.58 ± 0.60	<0.001 ***	2.96 ± 1.45	<0.001***
	Facebook	23 (5.30)	4.23 ± 1.41	<0.001 ***	4.17 ± 0.21	<0.001 ***	4.94 ± 0.29	<0.001 ***	2.02 ± 0.39	<0.001 ***	4.04 ± 0.47	<0.001***
	Line	12 (2.76)	2.17 ± 1.39	0.0222 *	2.60 ± 1.28	0.0135 *	2.63 ± 1.53	0.1312	1.42 ± 0.66	0.1874	1.88 ± 1.38	0.4279
	Messenger	1 (0.23)	1.00 ± 0.00	1.0000	1.00 ± 0.00	1.0000	1.00 ± 0.00	1.0000	1.00 ± 0.00	1.0000	1.50 ± 0.00	0.5714
	Others (Band)	1 (0.23)	3.80 ± 0.00	0.3180	3.60 ± 0.00	0.4424	5.00 ± 0.00	0.3548	1.00 ± 0.00	1.0000	5.00 ± 0.00	0.0599
SNS use by number of groups [c]	Non-user	250 (57.60)	1.03 ± 0.21	<0.001 ***	1.10 ± 0.56	<0.001 ***	1.14 ± 0.49	<0.001 ***	1.02 ± 0.12	<0.001 ***	1.03 ± 0.19	<0.001***
	Single app user	58 (13.36)	2.02 ± 1.47		2.28 ± 1.38		2.61 ± 1.52		1.34 ± 0.58		1.89 ± 1.36	
	2 apps user	109 (25.12)	2.95 ± 1.78		3.19 ± 1.26		3.82 ± 1.46		1.56 ± 0.62		2.94 ± 1.47	
	3 apps user	17 (3.92)	4.20 ± 1.38		4.15 ± 0.24		4.92 ± 0.34		1.95 ± 0.40		4.00 ± 0.50	

Note: Digital skill questions had response options ranging from 1 'Not at all true of me' to 5 'very true of me'. The Information Navigation skills were reversed since it contained negatively worded items. [a] Types of internet environment at home, Types of digital devices use, and Types of SNS use are not mutually exclusive. Only the number and percentage of answering "Yes" were reported to improve readability. Two-sample Wilcoxon signed-rank test was used to find difference between those who answered "Yes" and those who answered "No." [b] Two-sample Wilcoxon signed-rank test. [c] Kruskal–Wallis equality-of-populations rank test. *** p-value <0.001, ** 0.001 < p-value < 0.01, * 0.01 < p-value < 0.05, level of significance.

Supplementary Table S2. Distributions of Digital skills of the participants in the survey and Cronbach's Alphas for each domain (N = 434)

Digital skill domain	Total (Mean ± SD)	Internet access status (Mean ± SD)		
		Having access	No access	*p*-Value[a]
Frequency (Percent)	434 (100)	191 (44.01)	243 (55.99)	
Operational Skills (5 items) (α = 0.98)				
I know how to open downloaded files	1.84 ± 1.54	2.82 ± 1.84	1.08 ± 0.47	<0.001
I know how to download/save a photo I found online	1.85 ± 1.56	2.82 ± 1.86	1.09 ± 0.55	<0.001
I know how to use shortcut keys (e.g., CTRL-V)	1.72 ± 1.46	2.58 ± 1.83	1.04 ± 0.35	<0.001
I know how to open a new tab in my browser	1.73 ± 1.46	2.62 ± 1.83	1.02 ± 0.24	<0.001
I know how to bookmark a website	1.69 ± 1.42	2.53 ± 1.80	1.02 ± 0.24	<0.001
Total	1.77 ± 1.44	2.67 ± 1.77	1.05 ± 0.30	<0.001
Information Navigation skills (5 items) (α = 0.94)				
I find it hard to decide what the best keywords are to use for online searches	1.54 ± 1.20	1.98 ± 1.45	1.19 ± 0.80	<0.001
I find it hard to find a website I visited before	1.97 ± 1.58	2.98 ± 1.76	1.18 ± 0.75	<0.001
I get tired when looking for information online	2.03 ± 1.60	3.12 ± 1.73	1.18 ± 0.76	<0.001
Sometimes I end up on websites without knowing how I got there	1.99 ± 1.59	3.05 ± 1.75	1.16 ± 0.72	<0.001
I find the way in which many websites are designed confusing	1.98 ± 1.59	3.03 ± 1.77	1.16 ± 0.70	<0.001
Total	1.90 ± 1.36	2.83 ± 1.41	1.17 ± 0.72	<0.001
Social/ communication skills (5 items) (α = 0.98)				
I know which information I should and shouldn't share online	2.18 ± 1.69	3.40 ± 1.73	1.23 ± 0.80	<0.001
I know when I should and shouldn't share information online	2.15 ± 1.67	3.37 ± 1.73	1.19 ± 0.74	<0.001
I am careful to make my comments and behaviors appropriate to the situation I find myself in online	2.15 ± 1.69	3.41 ± 1.73	1.17 ± 0.74	<0.001
I know how to change who I share content with (e.g., friends, friends of friends or public)	1.91 ± 1.58	2.92 ± 1.83	1.12 ± 0.63	<0.001
I know how to remove friends from my contact lists	2.39 ± 1.80	3.72 ± 1.67	1.34 ± 1.06	<0.001
Total	2.16 ± 1.61	3.36 ± 1.62	1.21 ± 0.72	<0.001
Creative skills (5 items) (α = 0.85)				
I know how to create something new from existing online images, music or video	1.33 ± 0.75	1.69 ± 0.96	1.04 ± 0.31	<0.001
I know how to make basic changes to the content that others have produced	1.29 ± 0.67	1.62 ± 0.89	1.02 ± 0.18	<0.001
I know how to design a website	1.04 ± 0.21	1.07 ± 0.26	1.02 ± 0.16	<0.01
I know which different types of licenses apply to online content	1.24 ± 0.52	1.48 ± 0.62	1.04 ± 0.30	<0.001
I would feel confident putting video content I have created online	1.27 ± 0.70	1.58 ± 0.92	1.03 ± 0.29	<0.001
Total	1.23 ± 0.48	1.49 ± 0.59	1.03 ± 0.20	<0.001
Mobile Internet skills (2 items) (α = 0.88)				
I know how to download and install apps on mobile devices	1.95 ± 1.61	3.05 ± 1.83	1.09 ± 0.55	<0.001
I know how to keep track of the costs of mobile app use.	1.53 ± 1.08	2.14 ± 1.34	1.05 ± 0.37	<0.001
Total	1.74 ± 1.29	2.59 ± 1.50	1.07 ± 0.45	<0.001

Note: [a] Two-sample Wilcoxon signed-rank test. Each item had response options ranging from 1 'Not at all true of me' to 5 'very true of me'. The Information Navigation skills were reversed since it contained negatively worded items.
